# Surface nanobubbles on the carbonate mineral dolomite†

**DOI:** 10.1039/c8ra07952h

**Published:** 2018-10-16

**Authors:** Camilla L. Owens, Edgar Schach, Martin Rudolph, Geoffrey R. Nash

**Affiliations:** College of Engineering, Mathematics and Physical Sciences, University of Exeter EX4 4QF UK co308@exeter.ac.uk; Helmholtz Institute Freiberg for Resource Technology, Helmholtz-Zentrum Dresden-Rossendorf Chemnitzer Straße 40, 09599 Freiberg Germany

## Abstract

Surface nanobubbles are of wide interest to a number of research fields, ranging from mineral processing to metamaterials. Their formation on hydrophobic surfaces has long been confirmed but the factors controlling their size and location are less well understood. In this work we investigate, using non-contact atomic force microscopy, the properties of surface nanobubbles on the mineral dolomite under three aqueous solutions; water, depressant and collector. Nanobubbles were observed under all three conditions, but with the highest density observed under collector conditions. Analysis of the critical angle of the bubbles suggests that the collector does not affect the surface tension of the bubbles, but instead does affect their pinning, consistent with the observed increased density.

## Abstract 2

Analysis of surface nanobubbles on dolomite show that their pinning is affected by the surfactants using in mineral processing.

## Graphical abstract

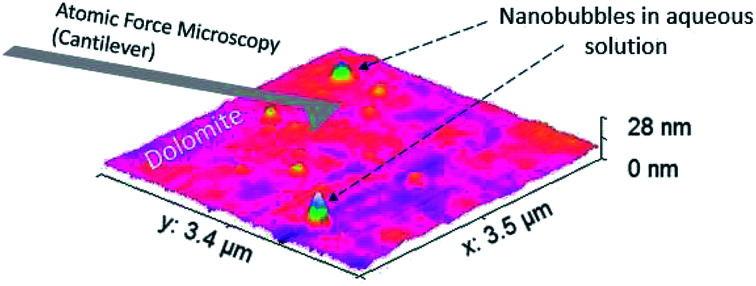

## Introduction

Since surface nanobubbles were first imaged in 2000,^1,2^ they have been of growing interest to research due to their long lived properties, with reported lifetimes as long as several hours.^3^ Surface nanobubbles are smaller, but have longer lifetimes than their bulk counterparts, and can be formed using electrolysis, to ethanol and water exchange, gas supersaturation and microwaves.^4–7^ Surface nanobubbles have been shown to form on a wide range of surfaces, ranging from pyrolytic graphite graphene to chalcopyrite and galena,^8–11^ and as well as being relevant to the understanding of colloids and surfaces, nanobubbles are thought to play an import role in processes ranging from cancer treatment to decompression sickness.^12,13^ Predicting and controlling the size and location of nanobubbles is therefore of interest to a wide range of fields,^14^ including in mineral processing, where bulk nanobubbles have been identified as improving the efficiency in flotation systems.^15^

In this paper we image nanobubbles on the surface of a carbonite mineral, dolomite, and investigate the effect of surfactants on their properties. Such surfactants are used in flotation processing systems to affect the hydrophobicity of the mineral surface, allowing separation of minerals.^16^ “Collectors” and “depressants” increase or decrease the hydrophobicity of the surface respectively. The surface behaviour of carbonate minerals is of significant interest to the mineral processing industry as carbonates form the constituent parts in many deposits,^16^ including those of rare earth element deposits.^17^ Complex mineralogy in many newly developed deposits makes extraction difficult, and an improved understanding of the role of nanobubbles in the flotation process could lead to more efficient extraction.

## Experimental

Non contact-atomic force microscopy (NC-AFM) was conducted on a Park Systems (South Korea) atomic force microscope XE100 located in Helmholtz Institute of Resource Technology Freiberg, Germany. The NC-AFM was combined with Raman Spectroscopy and an optical microscope to enable mineral identification and mapping. Images were produced in either 36 μm × 36 μm or 8 μm × 8 μm sizes. Nanobubbles were generated using previously described air water supersaturation method. Both Contact cantilever (Park systems nanotechnology solutions partner) PPP-CONTSCR 10M and ContAl-G Cantilever were used with a spring constant of 0.2 N m^−1^.^4^ The liquid cell used was of the same composition previous described by Rudolph and Peuker.^10^

The dolomite sample was analysed using X-ray diffraction and its composition confirmed with reference to the RTUFF database.^18^ High purity of the sample was calculated at 87.5%. The dolomite sample was set in epoxy resin then machine polished. For NC-AFM measurements water with KCl 10^−3^ mol L^−1^ background electrolyte and a collector solution (of combined fatty acids (betacol) and hydroxamates (AM810) in usual proportions for flotation) were prepared before measurements and cooled to 5 °C. This methodology was repeated with the water solution and depressant solution (combination of depressants from mineral processing). The exact composition of the depressant and collector is subject to non-disclosure. The liquid cell was filled by injecting up to 750 μL of collector, water or depressant solution using a clean disposable plastic nozzle attached to a pipette. The same clean disposable plastic nozzles have previously been used by Babel and Rudolph,^11^ with plastic nozzles also being used in other studies.^19,20^

The liquid was then heated to between 20 °C and 30 °C for nanobubble measurements in a temperature controlled room of 21 °C ± 1 °C. Between measurements the cantilever was cleaned by washing in distilled water then ethanol and again distilled water. The dolomite surface was polished with DiaPro 
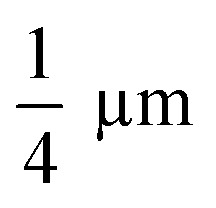
 diamond suspension by Struers to provide a fresh surface for each experiment. Subsequently, the surface was thoroughly washed with water, ethanol and water again to remove any residuals from the DiaPro 
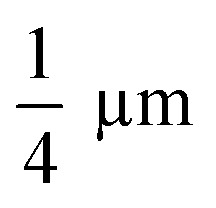
 suspension or other sources of contamination. The dolomite sample was then further cleaned with sonication before being rinsed with deionized water as a last step.

## Results and discussion

**Fig. 1:**
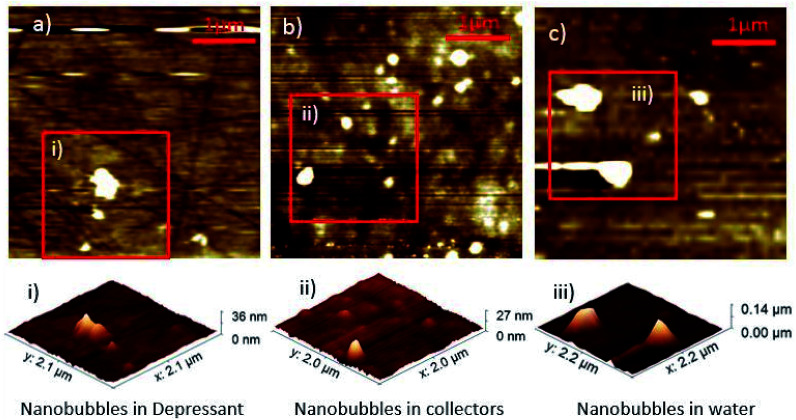
Nanobubble density differences with topographical AFM images of nanobubbles in depressant (a) collectors (b) and water (c). Each image is 4 μm × 4 μm across.

**Fig. 2:**
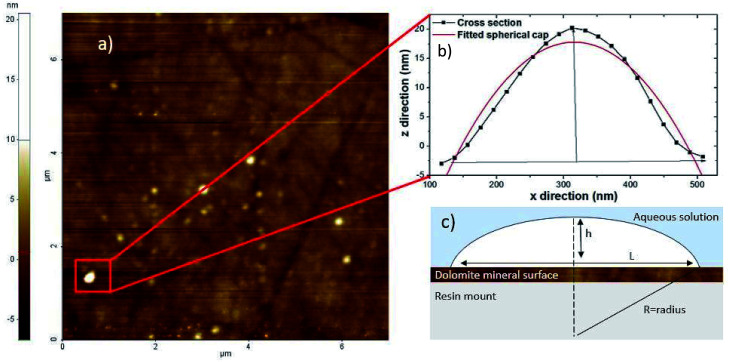
(a) 8 μm x 8 μm non contact atomic force microscopy (AFM) image of surface nanobubbles on the carbonate mineral dolomite (b) cross section of nanobubble height and width extracted from the non contact AFM image (c) diagram of height (*h*), length (*L*) and radius (*R*) with contact angle (*θ*) of the nanobubble. Diagram (c) after Lohse and Zhang.^14^

Nanobubbles were imaged on the surface of dolomite under water, collector and depressant conditions, as shown in Fig. 1. Image analysis from NC-AFM resulted in four good images, two images of nanobubbles in water, two images with nanobubbles in collector solution. To determine the density of the nanobubbles under different conditions, nanobubbles were identified from the images by comparing the phase and topography images from NC-AFM, which showed differences between nanobubbles and the mineral surface on the phase image. Nanobubbles were also identified by analysing their measured cross sections. Four cross sections of the nanobubbles were extracted and fitted to a spherical cap,^14^ as shown in Fig. 2, following the methodology proposed by Rangharajan *et al.*^21^ and Li *et al.*^22^ Fitting was conducted using non-linear least squares, with the height and lateral length as fitting parameters (see ESI† for more details). In total, 31 nanobubbles were identified from 63 possible candidates (ESI†) (nanobubbles had to show both phase difference and good fitting to cross sections to be identified as a nanobubble). The extracted nanobubble height ranged from, 73.9 nm to 7.8 nm, which compares to the value of 1.4 nm obtained for the root mean square of the surface roughness. Bubble density was calculated by dividing the number of bubbles by surface area, and under collector conditions the nanobubble density was 0.656 per μm^2^. In contrast the nanobubbles in water conditions had a bubble density of 0.342 nanobubbles per μm^2^, with depressants much lower at 0.0625 nanobubbles per μm^2^.

**Fig. 3:**
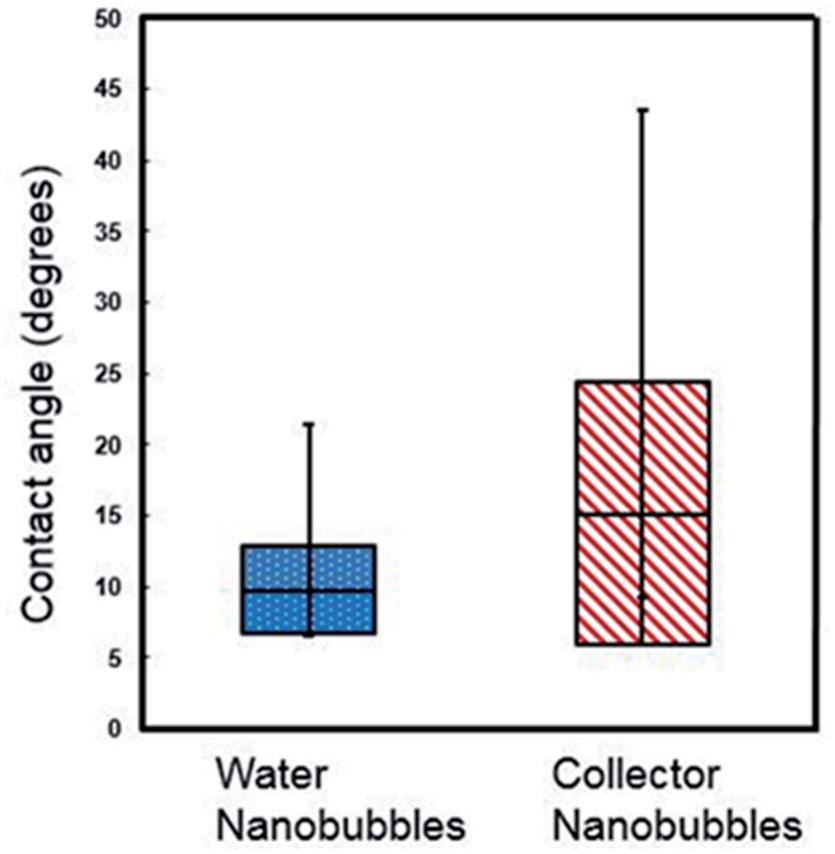
Box and whisker plot of contact angles of nanobubbles under collector and DI water conditions (the data was selected using fitting of a spherical cap model after Lohse and Zhang.^14^

Contact angles were extracted from the cross section fits, correcting for the size of the cantilever tip. In this study, a cantilever with a tip radius of 7 nm was used,^23,24^ and correction of the contact angle was calculated following methodology from Wang *et al.*^29^ Nanobubbles in the collector reagent scheme had an average contact angle of 9.74° with standard deviation ± 3.07, whereas those in water conditions had an average contact angle of 15.14° with standard deviation of ±9.27°, as plotted in box and whisker format in Fig. 3. The collector contact angle was the average of 21 nanobubble contact angles with the water being an average of 11 nanobubble values.

**Fig. 4:**
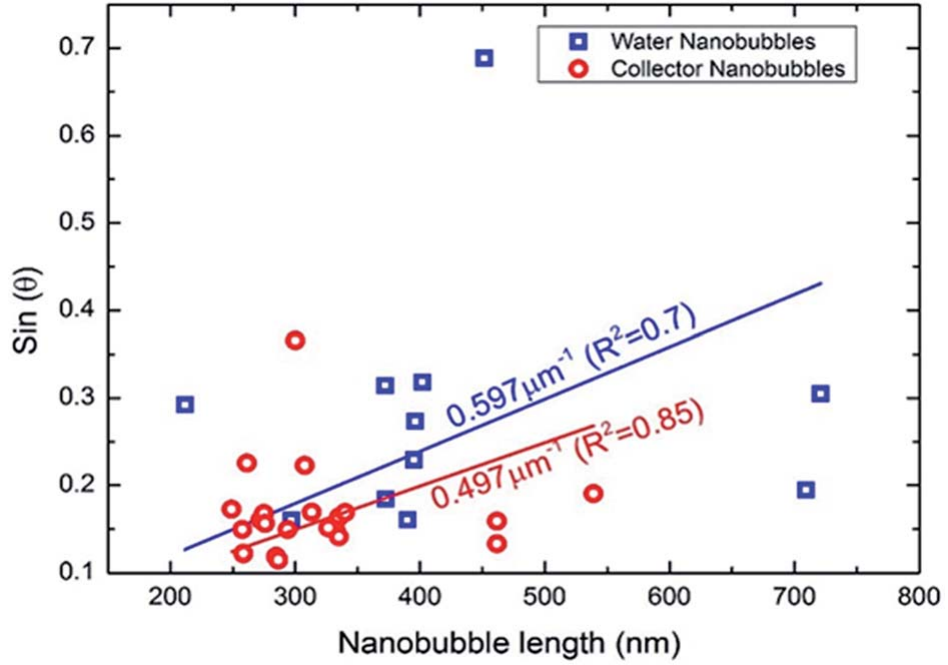
Sine of the contact angle as a function of lateral length under water and collector conditions.

The amount of supersaturation (*ξ*) of the liquid has also been proven to clearly effect both contact angle, *θ*, and the lateral length of the nanobubble, with Lohse and Zhang^14^ showing that for a (a fixed) gas oversaturation *ζ* > 0, there exists a stable equilibrium defined by:1
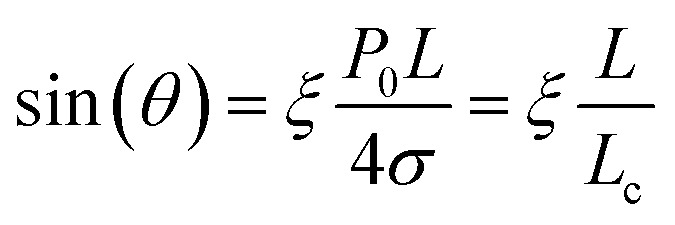
where *θ* is the contact angle, *L* the lateral length, and *L*_c_ the critical lateral extension (*L*_c_ = 4*σ*/*P*_0_ ≈ 2.84 μm),^14^ where *σ* is the surface tension and *P*_0_ the ambient pressure). Although reagents such as fatty acids have been shown to effect surface tension in macroscopic bubbles,^25,26^ the contact angle and therefore surface tension of nanobubbles does not necessarily appear to be effected by reagents.^27,28^ In Fig. 4, the sine of the contact angle is plotted as a function of the nanobubble length under both water and collector conditions. In both cases sin(*θ*) shows an approximately linear dependence on the length (the straight lines shown are linear fits to the data), consistent with eqn (1). Values of the ratio of *ξ*/*L*_c_ of 0.6 μm^−1^ and 0.5 μm^−1^, for nanobubbles in water and collector respectively, were extracted from gradient (eqn (1)) of the linear fits shown. Wang *et al.*,^29^ obtained a value of *ξ*/*L*_c_ of 2.9 μm^−1^, for nanobubbles induced in nanopits, which coupled with a value of *L*_c_ of 2.84 μm, led to an estimated oversaturation *ξ* of 8.2. In our case, taking the same value of *L*_c_, the gradient leads to an estimated oversaturation *ξ* of 1.7 under water conditions. The value of oversaturation obtained in this work therefore seems reasonable given the different methodologies in which nanobubbles were induced in these studies.

In addition, there is no significant difference between the values of *ξ*/*L*_c_ obtained under water and collector conditions. Assuming that the over-saturation and the ambient pressure where the same in both cases, this also implies that the surface tension was not affected by the collector. This is consistent with the thin film model of nanobubbles developed by Zhang *et al.*^27,30^ However, the difference in the density of bubbles observed under water and collector conditions suggests that the collector has affected the pinning of the bubbles, which in turn determines the lateral length. Previous work by Xiao *et al.*^31^ investigating the stability of nanobubbles under surfactants has shown with molecular dynamics simulations that the contact angle does not depend on pinning, whereas the density of nanobubbles does.^31^ This is consistent with earlier results by Mikhlin *et al.*^8^ who investigated nanobubbles at the surface of the sulphide mineral galena (PbS) and found that the number of nanobubbles increased after the surface had been pre-treated using a xanthate collector rather than with water. Tan *et al.*^32^ investigated the exact value of the pinning force by using an AFM tip to deform nanobubbles whilst imaging the mechanical response using total internal reflection fluorescence microscopy (TIRFM). The pinning strength varied between 5 mN m^−1^ to 20 mN m^−1^, with the variation attributed to chemical and physical heterogeneities of the surface. The relative smoothness of the mineral surface in this case indicates that it is chemical heterogeneities induced by the collector that affects the bubble pinning. Previous work by Xie^33^ has shown non uniform adsorption of xanthate on sphalerite caused differing regions of hydrophobicity on the mineral surface.

Finally, the increase in the observed number of nanobubbles under collector conditions is also consistent with macroscopic studies investigating micro flotation for minerals processing. Both Espiritu and Waters^16^ and Azizi and Larachi^34^ investigated micro flotation of dolomite under collector (hydroxamic acid and fatty acid). In Azizi and Larachi,^34^ 75.3% of dolomite was recovered (floated) with hydroxamic collector compared to 4.1% of dolomite was floated (recovered) under water conditions. Surface nanobubbles could therefore be playing an important part in froth flotation, although much work is required to quantify the significance of this contribution. Future work may focus on more minerals and reagent regimes, in particular rare earths were processing is of such importance to the viability of deposits.^17^

## Conclusions

Surface nanobubbles on dolomite, induced using the air water supersaturation method, were imaged using NC-AFM under water, collector and depressant surfactant conditions. The observed bubble density was highest under collector conditions, with 0.656 bubbles per μm^2^, compared to 0.342 nanobubbles per μm^2^ under water conditions. Analysis of the bubble contact angles, which were extracted by fitting a spherical cap to the nanobubbles, suggests that the collector does not affect the surface tension, but does affect their pinning. This is consistent with both the observed bubble density, but also with macroscopic flotation studies. These results there lay the foundation for an improved understanding of the role of nanobubbles in the flotation process.

## Conflicts of interest

There are no conflicts to declare.

## Supplementary Material

RA-008-C8RA07952H-s001
