# Correction: Surface nanobubbles on the carbonate mineral dolomite

**DOI:** 10.1039/c9ra90050k

**Published:** 2019-07-08

**Authors:** Camilla L. Owens, Edgar Schach, Martin Rudolph, Geoffrey R. Nash

**Affiliations:** College of Engineering, Mathematics and Physical Sciences, University of Exeter EX4 4QF UK co308@exeter.ac.uk; Helmholtz Institute Freiberg for Resource Technology, Helmholtz-Zentrum Dresden-Rossendorf Chemnitzer Straße 40 09599 Freiberg Germany

## Abstract

Correction for ‘Surface nanobubbles on the carbonate mineral dolomite’ by Camilla L. Owens *et al.*, *RSC Adv.*, 2018, **8**, 35448–35452.

The authors regret that a sentence in the original manuscript contained an error regarding the use of plastic nozzles. In order to avoid misleading readers, the sentence on page 35448 “The same clean disposable plastic nozzles have previously been used by Babel and Rudolph,^11^ with plastic nozzles also being used in other studies.^19,20^” should be given as “The same clean disposable plastic nozzles have previously been used by Babel and Rudolph.^11^”.

The Royal Society of Chemistry apologises for these errors and any consequent inconvenience to authors and readers.

## Supplementary Material

