# Supplementary material for: Surface nanobubbles on the carbonate mineral dolomite
Source: RSC Adv. 2018 Oct 16;8(62):35448–52. doi: 10.1039/c8ra07952h (PMC9087822; doi:10.1039/c8ra07952h)
Supplement: RA-008-C8RA07952H-s001 [file RA-008-C8RA07952H-s001.pdf]

Electronic Supplementary Material (ESI) for RSC Advances.
This journal is © The Royal Society of Chemistry 2018

Supporting information of

Surface nanobubbles on the carbonate mineral dolomite.

Camilla L. Owens[†*], Edgar Schach[‡], Martin Rudolph[‡], Geoffrey R. Nash[†].

[†]College of Engineering, Mathematics and Physical Sciences, University of Exeter, Exeter EX4 4QF, United Kingdom

[‡]Helmholtz Institute Freiberg for Resource Technology, Helmholtz-Zentrum Dresden-Rossendorf, Chemnitzer Straße 40, 09599 Freiberg, Germany.

[*]corresponding author

(a) Phase and cross section analysis of nanobubbles

Contamination at the surface of a material has the possibility for being mistaken for nanobubbles, due to this, each nanobubble in this manuscript was investigated to check they had been identified correctly. The nanobubbles had to show both phase difference and good fitting to cross sections, at a height above 8nm. This methodology followed work by Rangharajan *et al.,* [1] and Li *et al.,* [2]. Cross sections were fitted across four angles of the nanobubble. Figure 1 shows an example for the selection with R^2 fitting over a 0.8 for all four cross sections. If two of the cross sections had a $R^2 < 0.8$ then the nanobubble was discounted. Nanobubbles below 8nm in all four cross sections were also discounted, these are highlighted on figure 1 in black.

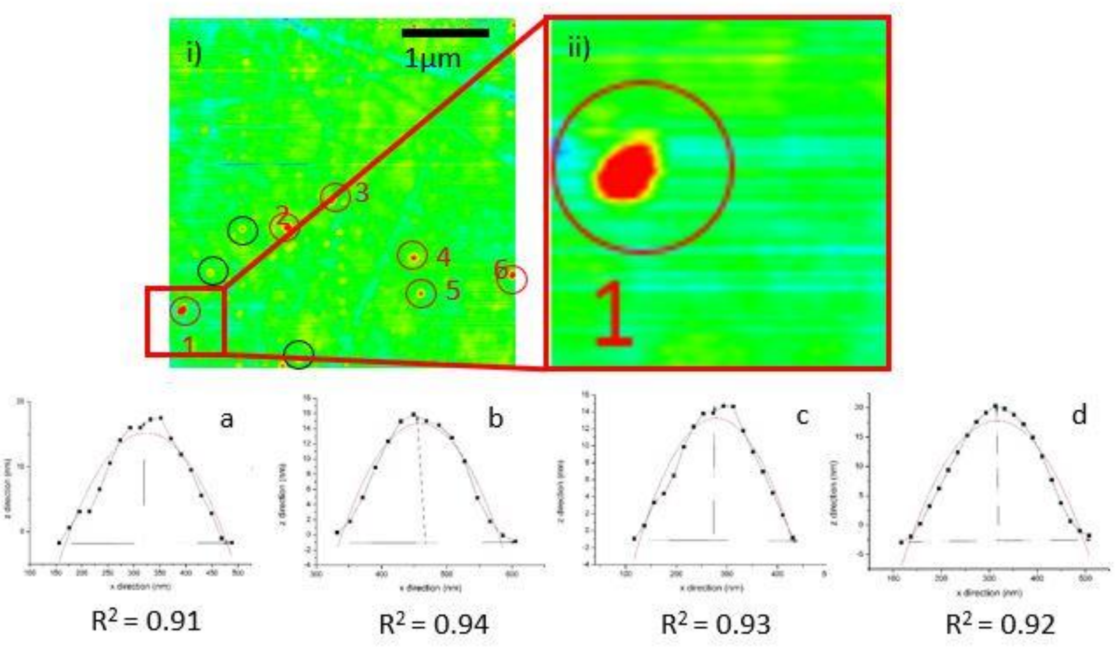

Figure 1. Nanobubble selection using cross sections. I) Nanobubbles on the surface of dolomite 4µm x 4µm, nanobubbles circled and numbered in red ii) enlarged section with nanobubble investigated in cross sections a,b,c,d. R^2 *values of a,b,c,d cross sections are shown below the figure.*

Nanobubbles were fitted to a spherical cap using Origin 8.6 Data Analysis graphing software using non-linear curve fit. All fitting was conducted in Origin 8.6 after exporting the cross sections from the atomic force microscopy image.

Comparison of topography and phase in AFM was conducted by lining up specific areas against each other, see figure 2.

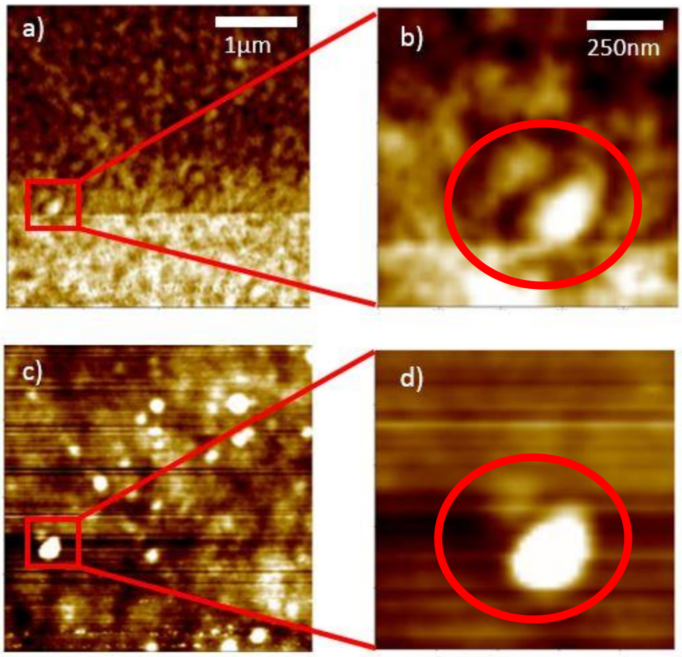

Figure 2. Topography and phase diagrams of nanobubbles in collector solution. a) and b) are phase diagrams for nanobubbles in collector conditions, with b) being an enlargement of image a). c) and d) are topographical images over the same area as a) and b) respectively.

Figure 2 shows that good agreement between the phase and topographical images of atomic force microscopy. Nanobubbles were selected if they appeared on both phase and topographical images.

(b) Cantilever tip radius correction

Cantilever tips have long been a known as an area of error for the width measurement of a nanobubble. Equations for the correction of nanobubble radius, diameter (L) and contact angle (θ) were taken from Wang *et al.,* [3].

$$R_c{}' = \frac{(D)^2+4H^2}{8H} - R_{tip} \quad [1]$$

$$L' = \sqrt{L^2 - 8HR_{tip}} \quad [2]$$

$$\theta' = 2\arctan(\frac{2H}{L'}) \quad [3]$$

R_c' is the corrected radius, L' is the corrected length (diameter) of the nanobubble and θ' is the corrected contact angle.

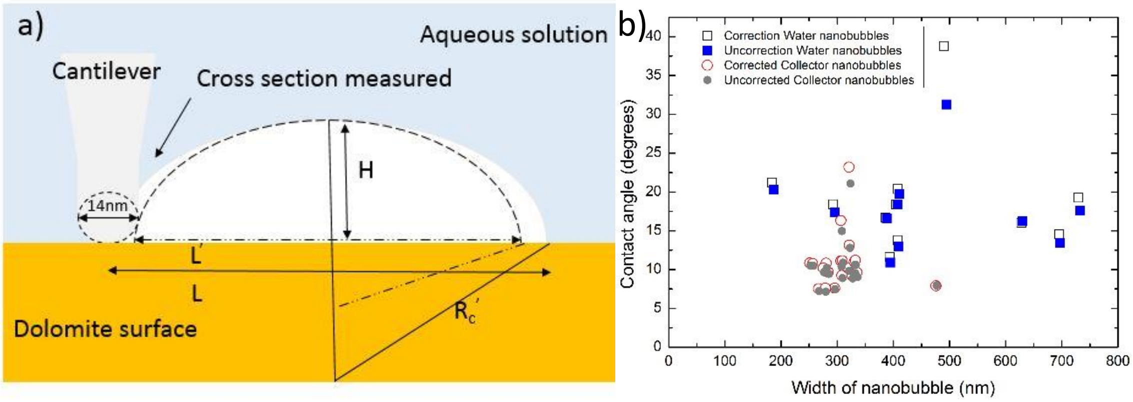

Figure 3 adapted from Wang et al., [3] Figure S7(a) adapted here to help in ease of the measurement. b) plot of width of nanobubbles against contact angle of both water and collector nanobubbles in corrected and uncorrected form.

Figure 3 (b) shows that the cantilever tip correction does not have a significant effect on the width and contact angles of the nanobubbles.

References

[1] K. K. Rangharajan, K. J. Kwak, A. T. Conlisk, Y. Wu and S. Prakash, Soft Matter, 2015, **11,** 5214-5223.

[2] D. Li, D. Jing, Y. Pan, W. Wang and X. Zhao, Langmuir, 2014, **30,** 6079-6088.

[3] Y. Wang, X. Li, S. Ren, H. T. Alem, L. Yang and D. Lohse, Soft matter, 2017, **13,** 5381-5388.
